# Food-based domestic violence and anemia among women in sexual unions in Nigeria: the effect of urbanization

**DOI:** 10.1057/s41271-024-00504-2

**Published:** 2024-07-12

**Authors:** Seun Mauton Ajoseh, Ridwan Islam Sifat, John Tasheyon Whesu

**Affiliations:** 1 Department of Sociology and Criminology & Law, University of Florida, Gainesville, FL 32603 USA; 2 School of Public Policy, University of Maryland, Baltimore County, Baltimore, MD 21250 USA; 3 Department of Sociology, Case Western Reserve University, Cleveland, OH 44106 USA

**Keywords:** Domestic violence, Food insecurity, Anemia, Sexual union, Rural–urban, Urbanization

## Abstract

In 2019, 1.74 billion people worldwide had anemia. In Nigeria, women of reproductive age are the most affected. Domestic violence affects the anemia prevalence, but few studies have examined the influence of urbanization on women in heterosexual unions (currently married, and cohabiting between). Using the social determinants of health framework, we argue that food-based violence and anemia vary among women residing in rural and urban areas. We used the Chi-square test and logistic regression to analyze the 2018 Nigeria Demographic and Health Survey records (n = 10,499). The study showed that anemia occurs more among women in rural (66%) than in urban (34%) areas. In rural areas, anemia was 29% higher among women who approved food-based domestic violence (OR: 1.29, CI 1.15–1.57) than those who did not. In urban areas, food-based domestic violence was not significantly associated with anemia. This study emphasizes the urgent need for culturally sensitive maternal health interventions aimed at re-orienting individuals and families on violence in rural areas.

## Key messages

- Anemia occurs more frequently among women in rural settings than in urban locations.
- Food-based domestic violence was associated with the incidences of anemia among women in rural areas but not significant in urban settings.
- Violence is normalized in rural areas, calling for an urgent need for culturally sensitive maternal health interventions.

## Introduction

Anemia remains a global public health challenge, particularly affecting reproductive-aged women [1, 2]. About half a billion women aged 15 to 49 are affected by anemia globally. In 2019, anemia affected 30% (539 million) of non-pregnant women and 37% (32 million) of pregnant women [3]. The World Health Organization (WHO) characterizes anemia by a reduced concentration of hemoglobin or a decrease in the quality or number of red blood cells [4]. Consequently, it diminishes the capacity of blood to carry oxygen to the body's tissues, leading to adverse health outcomes [5]. Women in developing countries present significant risks of anemia such as higher maternal mortality rates, limited physical and cognitive development, reduced productivity, and weakened immune responses [6, 7]. The burden is particularly severe in Sub-Saharan Africa due to the interplay of malnutrition, infectious diseases, inadequate health services, and socioeconomic disparities [8, 9].

In Nigeria, the prevalence of anemia among women of reproductive age is high and influenced by a combination of factors [10, 11], such as nutritional deficiencies, and iron deficiency in particular [12, 13], multiparity, and third trimester of pregnancy [13]. Nutritional deficiencies often result from inadequate dietary intake, poor dietary diversity, blood loss due to menstrual periods or childbirth, and gendered food insecurity due to exposure to domestic violence [14, 15]. There are two potential pathways in the literature between violence and nutrition [15]. First, food preparation, division, and lack of food may trigger violence [16, 17]. Second, food could be part of a broader set of violent practices influencing adverse nutritional outcomes [15]. For the second pathway, violence against women has direct nutritional effects, such as undernourishment, stress, depression, and adverse psychological and physiological outcomes which can indirectly affect nutrition [15, 18]. Living in a violent household can induce physiological changes that can exacerbate malnutrition [19], and harm health and well-being [20].

Previous studies have demonstrated that the experience of violence influences food insecurity and anemia. Globally, there is a consistently significant relationship between domestic violence and food insecurity in different regions [21]. Domestic violence can work through a mediation pathway (mental health and functioning, relationship quality, and alcohol use), and a moderation pathway (gender, and marital status) to influence food insecurity globally [21]. Thus, exposure to domestic violence is associated with nutrition and food insecurity [22, 23]. Consequently, domestic violence increases the risks of anemia among women [24–26]. In Nigeria, where patriarchal norms are deeply rooted, women in sexual unions are predisposed to the risk of domestic violence [27]. In the current study, sexual unions refer to sexual relationships between men and women, which is the only legal and recognized sexual union in Nigeria [28]. Strikingly, this violence against women is reportedly justified by a significant number of women themselves [29]. That is the women accept physical abuse under certain conditions such as burning food during preparation, refusing to have sex with the husband, arguing with the husband, going out without telling the husband, and neglecting the children [30]. As a result, domestic violence increases the risk of anemia among women in Nigeria [31]. Women with few or no resources of their own often experience abuse from their partners which makes them unable to purchase and consume foods that meet their dietary and nutritional needs [32].

Despite the known effects of domestic abuse on food insecurity and health, little is known about how it affects anemia in Nigerian women in sexual unions. A study conducted on family and clinical factors of domestic violence among pregnant women in Nigeria found that domestic violence was associated with anemia [31]. The authors of that study restricted the study population to 333 pregnant women who attended antenatal clinics. Using Nationally represented data, we examined the influence of domestic violence on anemia among women in sexual unions. Because domestic violence is colored by cultural context through the normalization of violence in the environment influencing the actual experience [31, 33], we used the justification of wife beating as a proxy for food-based domestic violence. Using the social determinant of Health framework, this study examines the rural versus urban dimension of the influence of food-based domestic violence, on anemia among women in sexual unions in Nigeria.

We used the social determinant of Health (SDOH) framework to guide the study. Social determinants of Health are the conditions in the environment where people are born, live, learn, work, play, worship, and age that affect a wide range of health, functioning, and quality-of-life outcomes and risks [34]. These are the mutable societal systems, the social resources, and hazards for health that societal systems control distribute, allocate, and withhold, which in turn, influence health outcomes [35]. The framers of SDH argued that social and political conditions give rise to social hierarchies, and based on individual’s positions in the hierarchies, their exposure and vulnerability to health conditions may vary [36]. Material (neighborhood quality), psychosocial (psychosocial stresses, social support), and behavioral and biological variables (nutrition, physical activity, tobacco use) mediate the social determinants [36]. The study aimed to examine the influence of living in rural and urban settings, which is an “intermediary determinant” of the SDH framework [36], in explaining the association of food-based domestic violence with anemia.

## Data and methods

### Data sources

The study analyzed the Nigeria Demographic and Health Survey records (NDHS, 2018). The NDHS is a nationally representative cross-sectional survey across the 36 states and the Federal Capital Territory of Nigeria. The survey covers topics such as demographic and socioeconomic indicators, maternal morbidity, and infant mortality, among others [37]. The survey utilized three-stage cluster sampling techniques based on a list of enumeration areas (EAs). The first stage is the selection of EAs from the Local Government Areas (LGAs), subdivisions of the 36 states, and the Federal Capital Territory (FCT). In the second stage, one EA is selected randomly from the list of localities from which households were chosen in the last stage. We selected forty-five households in every rural and urban cluster through systematic sampling techniques [37].

### Data extraction

The current study focused on women in heterosexual unions (currently married, and cohabiting). The outcome variable in this study is anemia status which we categorized in line with the WHO categorization and in previous studies. We categorized non-pregnant women with less than 12.0 g per deciliter (g/dl) hemoglobin count and pregnant women with less than 11.0 g/dl hemoglobin count as anemic. The main explanatory variable is ‘reporting wife beating for burning food,’ coded 1 as yes, and 0 as no. Other variables that measured domestic violence in NDHS (2018) were reporting wife beating for neglecting children, for arguing with the husband, for refusing to have sex with the husband, and goes out without telling the husband. We categorized each domestic violence as 0 for no, and 1 for yes. We use the place of residence coded as rural (0) and urban (1) to stratify the analysis [8, 38]. Using previous literature and the data available in DHS, we included the following covariates in the model: pregnancy status, breastfeeding status, level of education, use of mosquito nets, body mass index (BMI), source of drinking water, type of toilet facility, drug use status during pregnancy, pregnancy termination history, employment status, marital status, age, female autonomy, religion, and hormonal contraceptive use.

### Data analysis

We hypothesized that food-based violence influences the incidences of anemia among women in sexual unions. We presented the background characteristics of respondents and their anemic status using frequency and percentage distributions. We selected variables for multivariate analyses using bivariate Chi-square statistics. We eliminated variables above p > 0.05 in the Chi-square analysis from the multivariate analysis. For the multivariate analysis, we applied two models to examine the association between the explanatory variable on the outcome variable. The first model (Model 1) assessed the association using unadjusted odds ratio (uOR) of food-based violence only; the second model (Model II) included all the covariates to control for the influence to generate adjusted odds ratios (aOR). We estimated the probability values associated with uORs and aORs at the 5% significance level and 95% confidence intervals. Because the study utilized publicly available data, it does not need the ethical approval and consent of participants. Permission to download and use the dataset comes from https://www.dhsprogram.com.

## Results

### Background characteristics of the respondents

Table 1 shows the percentage distribution of background characteristics of respondents by their anemic status. About 15% of respondents reported food-based violence, and 62% of the women live in rural areas (Table 1). Also, the result indicates that food-based domestic violence, household wealth index, pregnancy status, residence type, level of education, region, body mass index (BMI), sources of drinking water, type of toilet facilities, drug use during pregnancy, total children ever born, employment status, maternal age, female autonomy, religion, hormonal contraceptive use, domestic violence for child neglect, domestic violence for arguing with husband, domestic violence for sex-related matters, and domestic violence for going out without informing their husbands were associated with anemic status (P < 0.05).

**Table 1:** Percentage distribution of women’s background characteristics by anemic status, NDHS 2018

	Anemic status			
Variables	Not anemic	Anemic	Total	p value
*Food-Based Domestic Violence*				< 0.0001
No	3,797 (87.61)	5,105 (83.01)	8,902 (84.91)	
Yes	537 (12.39)	1,045 (16.99)	1,582 (15.09)	
*Household Wealth Index*				< 0.0001
Poorest	699 (16.11)	1,390 (22.57)	2,089 (19.90)	
Poorer	834 (19.22)	1,327 (21.55)	2,161 (20.58)	
Middle	910 (20.97)	1,335 (21.68)	2,245 (21.38)	
Richer	953 (21.96)	1,216 (19.74)	2,169 (20.66)	
Richest	944 (21.75)	891 (14.47)	1,835 (17.48)	
*Pregnancy Status*				< 0.0001
No/unsure	3,800 (87.56)	5,217 (84.71)	9,017 (85.88)	
Yes	540 (12.44)	942 (15.29)	1,482 (14.12)	
*Breastfeeding Status*				0.568
No	2,854 (65.76)	4,017 (65.22)	6,871 (65.44)	
Yes	1,486 (34.24)	2,142 (34.78)	3,628 (34.56)	
*Residence Type*				< 0.0001
Rural	2,489 (57.35)	4,041 (65.61)	6,530 (62.20)	
Urban	1,851 (42.65)	2,118 (34.39)	3,969 (37.80)	
*Level of Education*				< 0.0001
No Education	1,520 (35.02)	2,678 (43.48)	4,198 (39.98)	
Primary	788 (18.16)	1,044 (16.95)	1,832 (17.45)	
Secondary	1,524 (35.12)	1,979 (32.13)	3,503 (33.37)	
Higher	508 (11.71)	458 (7.44)	966 (9.20)	
*Respondents Slept Under a Mosquito Bed*				0.155
No	2,046 (47.14)	2,817 (45.74)	4,863 (46.32)	
Yes	2,294 (52.86)	3,342 (54.26)	5,636 (53.68)	
*Region*				< 0.0001
North-central	839 (19.33)	1,071 (17.39)	1,910 (18.19)	
North-east	757 (17.44)	1,119 (18.17)	1,876 (17.87)	
North-west	1,033 (23.80)	1,563 (25.38)	2,596 (24.73)	
South-east	459 (10.58)	880 (14.29)	1,339 (12.75)	
South-south	476 (10.97)	701 (11.38)	1,177 (11.21)	
South-west	776 (17.88)	825 (13.40)	1,601 (15.25)	
*Body Mass Index (BMI)*				< 0.0001
Underweight	341 (7.87)	618 (10.04)	959 (9.14)	
Normal	2,427 (55.99)	3,888 (63.16)	6,315 (60.19)	
Overweight	1,567 (36.15)	1,650 (26.80)	3,217 (30.66)	
*Sources of Drinking Water*				0.017
Improved	2,630 (61.42)	3,580 (59.08)	6,210 (60.05)	
Unimproved	1,652 (38.58)	2,480 (40.92)	4,132 (39.95)	
*Type of Toilet Facility*				< 0.0001
Improved	1,903 (44.44)	3,105 (51.24)	5,008 (48.42)	
Unimproved	2,379 (55.56)	2,955 (48.76)	5,334 (51.58)	
*Drug use During Pregnancy*				0.001
No	2,580 (80.22)	3,842 (83.14)	6,422 (81.94)	
Yes	636 (19.78)	779 (16.86)	1,415 (18.06)	
*Ever Terminated Pregnancy*				0.853
No	3,645 (83.99)	5,181 (84.12)	8,826 (84.07)	
Yes	695 (16.01)	978 (15.88)	1,673 (15.93)	
*Total Children Ever Born*				0.012
1–2 children	1,496 (34.47)	2,078 (33.74)	3,574 (34.04)	
3–5 children	1,701 (39.19)	2,300 (37.34)	4,001 (38.11)	
6+ children	1,143 (26.34)	1,781 (28.92)	2,924 (27.85)	
*Employment Status*				< 0.0001
Unemployed	1,122 (25.85)	1,878 (30.49)	3,000 (28.57)	
Employed	3,218 (74.15)	4,281 (69.51)	7,499 (71.43)	
*Marital Status*				0.498
Married	4,158 (95.81)	5,917 (96.07)	10,075 (95.96)	
Cohabitation	182 (4.19)	242 (3.93)	424 (4.04)	
*Maternal Age*				0.035
15–24 years	824 (18.99)	1,284 (20.85)	2,108 (20.08)	
25–34 years	1,784 (41.11)	2,532 (41.11)	4,316 (41.11)	
35+ years	1,732 (39.91)	2,343 (38.04)	4,075 (38.81)	
*Female Autonomy*				< 0.0001
Not autonomous	2,510 (57.83)	3,861 (62.69)	6,371 (60.68)	
Autonomous	1,830 (42.17)	2,298 (37.31)	4,128 (39.32)	
*Religion*				< 0.0001
Christianity	2,150 (49.54)	2,745 (44.57)	4,895 (46.62)	
Islam	2,147 (49.47)	3,369 (54.70)	5,516 (52.54)	
Traditional/others	43 (0.99)	45 (0.73)	88 (0.84)	
*Hormonal Contraceptive Use*				< 0.0001
No	3,767 (86.80)	5,703 (92.60)	9,470 (90.20)	
Yes	573 (13.20)	456 (7.40)	1,029 (9.80)	
*Domestic Violence for Child Neglect*				< 0.0001
No	3,505 (80.83)	4,627 (75.22)	8,132 (77.54)	
Yes	831 (19.17)	1,524 (24.78)	2,355 (22.46)	
*Domestic Violence for Arguing with Husband*				< 0.0001
No	3,555 (81.99)	4,696 (76.41)	8,251 (78.72)	
Yes	781 (18.01)	1,450 (23.59)	2,231 (21.28)	
*Domestic Violence for Sex*				< 0.0001
No	3,550 (81.93)	4,652 (75.77)	8,202 (78.32)	
Yes	783 (18.07)	1,488 (24.23)	2,271 (21.68)	
*Domestic Violence for Going Out*				< 0.0001
No	3,514 (81.08)	4,591 (74.69)	8,105 (77.33)	
Yes	820 (18.92)	1,556 (25.31)	2,376 (22.67)	

### Multivariate result

Table 2 shows the relationship between food-based domestic violence and anemia by residence among women in sexual union. Model I (the unadjusted model) showed that, among women in rural areas, food-based domestic violence is significantly related to being anemic. Compared to women not reporting food-based domestic violence, women who reported being beaten when the food gets burnt were 38% (uOR = 1.38; 95% CI 1.21–1.57) more likely to be anemic, than non-anemic. When we controlled for other covariates for in Model II, there was a reduction in the odds of being anemic, although the relationship was still significant. Thus, women who reported beating for the food gets burnt, compared to those who are not, are 29% (aOR = 1.29; 95% CI 1.15–1.57) more likely to be anemic, than non-anemic. In urban areas, the results presented in Model I showed that the reporting beating does not predict the odds of being anemic. Similarly, in Model II, food-related gender-based violence is not significantly related to anemic status.

**Table 2:** Logistic Regression showing the association between the food-related gender-based V=violence and anemia status of women in sexual union in Nigeria, stratified by residential areas

	Rural		Urban	
	Model I	Model II	Model I	Model II
Variable	uOR (95% CI)	aOR (95% CI)	uOR (95% CI)	aOR (95% CI)
*Food-Based Domestic Violence (Ref: No)*				
Yes	1.38 (1.21–1.57) ***	1.29 (1.15–1.57) *	1.23 (0.97–1.58)	0.68 (0.44–1.05)
*Wealth Index (Ref: Poorest)*				
Poorer		0.83 (0.70–0.98) *		1.17 (0.74–1.86)
Middle		0.80 (0.66–0.98) *		0.99 (0.65–1.52)
Richer		0.75 (0.58–0.97) *		0.90 (0.58–1.39)
Richest		0.60 (0.42–0.84) **		0.77 (0.48–1.23)
*Pregnancy Status (Ref: No)*				
Yes		1.24 (1.04–1.47) *		1.27 (1.02–1.59) *
*Education (Ref: No Education)*				
Primary		0.85 (0.70–1.02)		1.03 (0.78–1.37)
Secondary		0.97 (0.80–1.20)		0.89 (0.68–1.16)
Higher		0.85 (0.59–1.23)		0.86 (0.62–1.19)
*Region (Ref: North Central)*				
North-east		0.79 (0.64–0.97) *		0.66 (0.48–0.91) *
North-west		0.70 (0.57–0.85) **		0.63 (0.47–0.84) **
South-east		1.84 (1.37–2.46) ***		2.13 (1.57–2.88) ***
South-south		1.80 (1.41–2.31) ***		1.27 (0.90–1.79)
South-west		1.18 (0.90–1.53)		1.12 (0.87–1.45)
*Body Mass Index (Ref: underweight)*				
Normal weight		0.91 (0.75–1.10)		0.94 (0.69–1.29)
Overweight/obese		0.66 (0.52–0.83) **		0.70 (0.50–0.98)
*Water source (Ref: Improved)*				
Unimproved		0.99 (0.87–1.12)		0.92 (0.78–1.10)
*Toilet Facilities (Ref: Unimproved)*				
Improved		1.08 (0.93–1.24)		1.05 (0.86–1.29)
*Drug Use during Pregnancy (Ref: No)*				
Yes		0.85 (0.72–1.00) *		0.92 (0.78–1.10)
*Children Ever Born (Ref: 1–2 Children)*				
3–5 children		0.97 (0.82–1.16)		1.02 (0.84–1.24)
6+ children		1.05 (0.84–1.31)		1.25 (0.94–1.66)
*Employment Status (Ref: Unemployed)*				
Employed		0.86 (0.74–0.98) *		0.98 (0.81–1.18)
*Maternal Age (Ref: 15–24 Years)*				
25–34 years		1.02 (0.85–1.23)		1.10 (0.87–1.40)
35+ years		0.99 (0.78–1.25)		1.08 (0.80–1.44)
*Female Autonomy (Non-autonomous)*				
Autonomous		0.87 (0.75–1.01)		0.95 (0.81–1.13)
*Religion (Ref: Christianity)*				
Islam		1.49 (1.22–1.81) ***		1.09 (0.87–1.35)
Traditionalist/others		0.49 (0.27–0.88) *		1.58 (0.50–6.02)
*Hormonal Contraceptive (Ref: No)*				
Yes		0.67 (0.53–0.84) ***		0.68 (0.55–0.85) **
*Domestic Violence for Going Out (Ref: No)*				
Yes		1.08 (0.83–1.40)		1.70 (1.13–2.56) *
*Domestic Violence for Child Neglect (Ref: No)*				
Yes		0.97 (0.73–1.27)		0.90 (0.59–1.37)
*Domestic Violence for Arguing with Husband (Ref: No)*				
Yes		0.95 (0.73–1.23)		0.92 (0.61–1.38)
*Domestic Violence for Sex (Ref: No)*				
Yes		1.07 (0.84–1.36)		1.54 (1.06–2.23) *

*Ref* - Reference category

*p < 0.05, **p < 0.01, ***p < *0.001*

## Discussion

The study found an intricate relationship between food-based domestic violence and anemia among women in heterosexual relationships in Nigeria. It shed light on underlying socio-cultural factors that intensify disparities in nutritional health. The results highlight how the presence of gender-based violence is not solely linked to actual instances of physical harm during food preparation but rather its normalization within certain cultural contexts. This distinction is crucial as it reveals a complex connection between societal norms regarding gender-based violence and the impact on women's health, particularly with regard to anemia.

This study's distinction between the occurrence of actual gender-based violence and the societal acceptance of such violence provides valuable insights into the impact of gender norms on experiences related to food insecurity and health. When women perceive gender-based violence as an acceptable response to actions like burning food, it can lead to increased stress and psychological distress, both of which are known factors that affect nutritional status and risk for anemia [39, 40]. This connection highlights how deeply embedded societal attitudes toward gender-based violence intersect with health outcomes for women. It emphasizes the need to address these entrenched beliefs in order to improve overall well-being among women.

Our research findings have shown the profound and far-reaching impact of food insecurity and gender-based violence on women's nutritional status. Building on existing knowledge, our unique contribution emphasizes the critical influence of normative beliefs regarding gender-based violence in shaping food preparation practices and contributing to anemia prevalence. This revelation underscores the need for interventions targeting Nigerian women's anemia rates to not only focus on improving nutrition and healthcare access but also will require actively addressing damaging gender norms that perpetuate violence.

Addressing food-based domestic violence and anemia in Nigeria demands a comprehensive and multifaceted approach. From a policy perspective, it is essential to develop holistic policy strategies that not only focus on nutritional health programs but also integrate gender equity and women's empowerment at their core. In addition, there is a crucial need for culturally sensitive educational campaigns aimed at shifting societal perspectives on gender-based violence, thereby impeding its detrimental health impacts. Empowering women by enhancing their autonomy and access to resources such as food and healthcare can effectively alleviate the repercussions of gender-based violence on anemia. These interventions must be guided by a social determinant of health framework that recognizes the interplay of social systems and resources, environment, and politics in shaping women’s health outcomes.

### Limitations

First, the NDHS data are prone to reporting and recall bias. Second, the main explanatory variable measures the belief system of the women on domestic violence as a proxy for the experience of domestic violence among the women. However, the perception of people on gender-based violence influences the experience of gender-based violence [32]. Future studies could better examine the relationship between gendered food insecurity and Anemia among reproductive-aged women in the urban slums, and the cultural context of violence in rural areas.

## Conclusions

Anemia is still prevalent among Nigerian women in sexual unions. Anemia is more prevalent among women in rural areas than in urban settings. Food-based domestic violence was associated with an increased prevalence of anemia among women in sexual unions in rural but not in urban areas. Thus, we recommend cultural reorientation of gender-based violence among men and women by the National Orientation Association, familial relationships, and other stakeholders in rural areas. To effectively pursue the United Nations' Sustainable Development Goal 2 (Zero Hunger) and Goal 3 (Good health and well-being), it is imperative to address gender-based violence in rural areas of sub-Saharan Africa. This is crucial to improve access to food and enhance food security for women and their children, thereby reducing the risk of anemia.

## Data Availability

Data will be available upon a request.
